# The Effect of Rosa Damascena Extract on Primary Dysmenorrhea: A Double-blind Cross-over Clinical Trial

**DOI:** 10.5812/ircmj.14643

**Published:** 2014-01-05

**Authors:** Soheila Bani, Shirin Hasanpour, Zeinabalsadat Mousavi, Parvin Mostafa Garehbaghi, Morteza Gojazadeh

**Affiliations:** 1 Department of Midwifery, School of Nursing and Midwifery¸ Tabriz University of Medical Sciences, Tabriz, IR Iran; 2 Department of Obstetric and Gynecology, Tabriz University of Medical Sciences, Tabriz, IR Iran; 3 Department of Medicine, Tabriz University of Medical Sciences, Tabriz, IR Iran

**Keywords:** Dysmenorrhea, Rosa, Mefenamic Acid

## Abstract

**Background:**

Dysmenorrhea is one of the most common types of cyclic pain that affects 50% of women and girls in their menstrual ages. Because of the side-effects and contraindications of chemical medicines, using herbs has been investigated in treating dysmenorrhea.

**Objectives:**

The aim of this study was to determine the effect of Rosa damascena extract on primary dysmenorrhea among the students of Kowsar dormitory in Tabriz University of Medical Sciences.

**Materials and Methods:**

This study was performed in Iran on 92 single 18-24 year old students with BMI :19-25 and obtaining pain intensity score of 5-8 in Visual Analogue Scale that were randomly classified and included in two groups of 46 persons. The participants received two capsules of Mefenamic Acid and Rosa damascena with the similar physical properties in two consecutive cycles per 6 hours for 3 days in a cross-over form. The data were collected through the questionnaire of demographic characteristics and check-list of visual analogue scale. Descriptive statistics and repeated measurement test and independent samples t test by using SPSS (13/win) were used in order to determine and compare the effects of two drugs on dysmenorrheal pain intensity of the groups.

**Results:**

There was a significant difference between the average of pain intensity at different hours of measurement in each group after the end of first cycle and second cycle (P < 0.001). There was no significant difference between the average of pain intensity in two groups in the first cycle (P = 0.35) and second cycle (P = 0.22).

**Conclusions:**

In this study¸ Rosa damascena and Mefenamic acid had similar effects on pain intensity of primary dysmenorrhea . With further studies, Rosa damascena which has no chemical side effects¸ can be suggested for treating primary dysmenorrhea.

## 1. Background

Dysmenorrhea is one of the most common types of cyclic pain that affects 50% of women and girls in their menstrual age and it is in two types of primary and secondary dysmenorrhea .The Primary type usually begins with ovulation and sometimes continues until the fifth decade of life (1).

In a study, the prevalence of no , mild, moderate, and severe menstrual pain in Iran, was 10%, 41%, 28%, and 22%, respectively (2). Intensive dysmenorrhea causes the individuals absence from the work or education. In the US alone, it is estimated that the annual economic loss is 600 million work hours and two billion dollars. Women, who continue to work or attend classes, have been shown to have lower work output during their dysmenorrhea (3, 4).

Primary dysmenorrhea is attributed to the increasing of endometrial prostaglandins. Treatments for dysmenorrhea are: non-steroidal anti-inflammatory drugs, hormonal medications and non-medical treatments such as acupuncture and trans-electrical nerve stimulation (TENS). Nowadays, the most common treatment is inhibitors of prostaglandin synthesis that is unfortunately forbidden for peptic ulcer disease. On the other hand, nausea, indigestion, peptic ulcer, and diarrhea are some of the side-effects of these treatments (5).

Because of the side-effects and contraindications of chemical medicines, using herbs such as fennel, cinnamon, and valerian has been investigated in treating dysmenorrhea (6, 7).

Rosa damascena commonly known as Damask rose is one of species of Rosaceae family, that is grown all around the world especially Iran. They are principally cultivated for using in perfume and food industry (8). The Rosa damascene has also been used for medicinal purposes (9). Various products and isolated constituents from flowers, petals and hips (seed-pot) of this plant have been studied in a variety of in vivo and in vitro studies and have been shown to have anti-hypnotic (10), Anti-convulsive (11), anti-HIV (12), Anti- diabetic , (13) and cardiovascular (14) modulating effects. Human studies have reported hypersensitivity and gastrointestinal side effects such as urticaria, nausea, vomiting, diarrhea and gastrointestinal discomfort for Rose hip.

Because of the natural essence of Rosa damascene extract, its high cultivation and consumption in Iran (15), analgesic and anti-inflammatory properties in various studies (10, 16-20), it seems that this extract can be helpful in treating painful menstruation.

## 2. Objectives

According to high prevalence of dysmenorrhea and its negative effects on social and individual life, this study was done in order to identify the effect of Rosa damascene extract on primary dysmenorrhea in female students of dormitory of Tabriz University of Medical Sciences in 2012.

## 3. Materials and Methods

This study was a double-blind cross-over randomized clinical trial that was performed on 92 students in Kowsar Dormitory of Tabriz University of Medical Sciences, Tabriz, Iran, from September 2012 to February 2013. The inclusion criteria of participating in this study were: age between 18 and 24, being single, BMI = 19-25, getting pain intensity score of 5-8 in visual analogue scale, filling written consent, no history of abdominopelvic surgery, not being prohibited from taking herbs and non-steroidal anti-inflammatory drugs (e.g. renal, hepatic or gastrointestinal disease). The exclusion criteria of participating were: occurrence of stressful event (e.g bereavement), using drugs which might interact with NSAIDs, using oral contraceptive pills and lack of compliance.

Our research society was one of the populous dormitories of Tabriz University of Medical Sciences (Kowsar Dormitory). After getting permission from the Ethics Committee (Ethical code 9159.date 27/6/2012), the interested students were invited to participate in this study by giving information to them. The goals of the study were explained to them. Then after getting informed consent from them and for Confidentiality of participant’s information, they were instructed to refrain from writing their names on the questionnaires.

Volunteer students with primary dysmenorrhea were given a questionnaire consisting of individual information, menstruation, and medical history in order to choose the samples and check list of visual analogue scale to fill it, in their next menses. The primary outcome was the pain intensity after the end of first cycle. For the sample size calculation we used findings from similar studies in comparable populations (21). Thus, the number of participants required to detect a standardized difference in pain intensity of 1.5 between groups, with 80% power using a cut-off score for statistical significance of 0.05 and with consideration 10% follow up lost ,was 92 (46 per group). Calculations for determining the sample size was done by the statistical software "Power & sample size".

From 124 students who volunteered to participate in the study and fill the checklists, 18 were excluded due to lack of inclusion criteria (one had BMI> 25, 3 had BMI < 19, 8 were married, four had less pain severity than 5 on VAS and 2 had more pain severity than 8 on VAS) and 106 students were selected with convenience sampling method (Flowchart of participants), according to eligibility criteria of this study. The random allocation was done by www. Random.org site with blocking method (size of blocks: 4 and 6) .So, samples were allocated in two groups randomly. The assistant put the drugs of each number in an envelope with the respective number written on the envelope. (The capsules of Rosa damascena extract were produced with the same appearance, color and odor as Mefenamic acid capsules) and 92 envelopes were given to the researcher to pass them to the corresponding participants.

The validity and reliability of pain intensity of visual analog scale (VAS) have been proved (22). To assess validity of the questionnaire in this study, we used qualitative content analysis. Questionnaires were sent to ten specialists in the field of the study and the material was revised and improved according to the feedbacks received. In order to gain reliability of visual analog scale (VAS) in this study, Test-retest method with 1 month interval was used in which 15 students from each group were asked to record the intensity of dysmenorrhea at the start of menstrual pain for two consecutive cycles. The correlation coefficient was calculated to be 85%.

The drugs consisted of: Mefenamic acid capsule 250mg, produced by Razak pharmaceutical company of Iran and Rosa damascena extract capsule 200mg produced by Yashil pharmaceutical company of Tabriz. Winther et al found that the effective dose of damascena when used as dried powder is 5 g per day (21), which is calculated as 15% of the extract. In case of providing the extract form, it is almost 750 mg per day. In present study it was used as 200 mg capsules every 6 hours. Mostafa-Gharabaghi et al. also used 800 mg of Rosa damascene extract daily to evaluate the effect of Rosa damascene on cesarian postoperative pain (18).

In order to produce capsules of Rosa damascene extracts, at first the dried fruits of Rosa damascene were changed to powder by mechanical grinder and then the extraction has been made by ethanol 70% using the method of maceration and extraction was repeated 3 times and each time for 5 hours. The extract was dried by rotary evaporator under reduced pressure. The dried extracts were kept in refrigerator below zero until using them and then they were used to produce capsules. The participants randomly received a drug in first cycle. During the study, the participants and the researcher were unaware of the drug. The participants were supposed to take one capsule every 6 hour at the first 3 days of menstruation. In the second cycle, the drugs were changed. The participants recorded the pain intensity exactly at the time of menstrual pain (before taking the drug) and in 1-2-3-6-12-24-48-72 hours after starting the menstrual pain.

The data obtained from the study were used in order to survey the pain intensity changes between two groups by using of descriptive statical methods (percent¸ frequency and standard deviation ± average) before and after cross-over. Repeated Measurement of anova test was used in the cases of having normal distribution and Friedman Test was used otherwise. Independent samples T-test was used in order to survey the quantitative variables such as age, BMI and between two groups. In this study we used SPSS (version13) and P < 0.05 was considered significant statistically.

## 4. Results

In the present study, two groups of 46 persons participated (92 persons). All of them entered the statistical analysis and no sample loss happened. Some of the characteristics of the participants of two groups exist in Table 1. There was no significant difference between two groups in comparing the characteristics of two groups from the viewpoint of age, BMI, age of beginning dysmenorrhea, menstrual pain intensity before the study, and the age at menarche (Table 1).Statistical analysis was done two times, before and after the cross-over. In order to compare the changes of pain intensity in two groups in specific times, Repeated Measurement of ANOVA was used.

**Figure 1.**
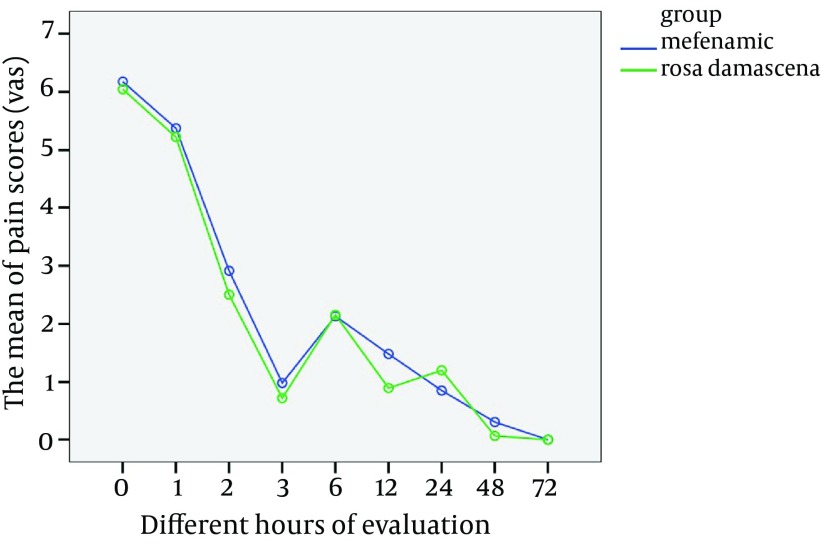
The comparison of Pain Scores in Different Hours of Evaluation After the End of First Cycle in Two Groups

The changes of pain intensity in two groups in specific times were measured after the end of first cycle (Table 2). There was a significant difference in comparing the average of pain intensity in different hours of measurement (pre and post treatment) in each group (P < 0.001); as the pain intensity decreased in each group .But there was no significant difference in comparing the average of pain intensity between two groups of Rosa damascena extract and Mefenamic acid (P = 0.35) (Figure 1). 

**Figure 2.**
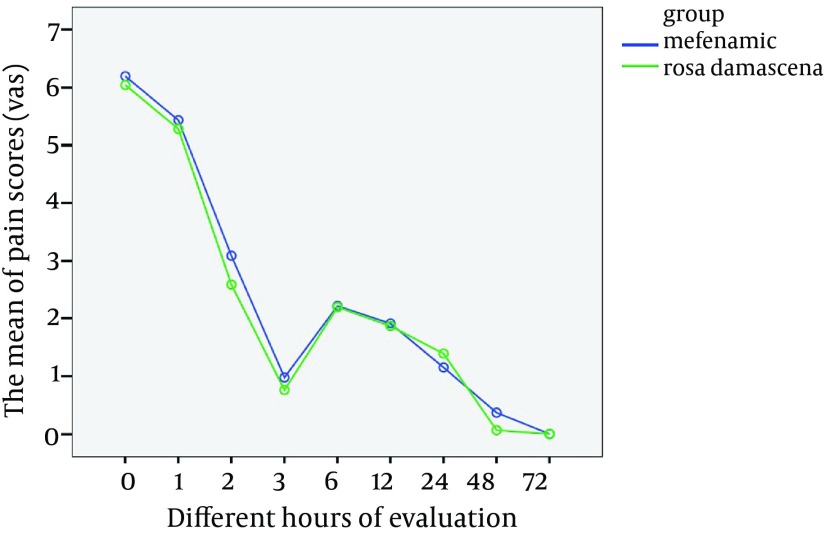
The Comparison of Pain Scores in Different Hours of Evaluation After the End of Second Cycle in Two Groups

After the end of the second cycle, the changes of pain intensity in two groups in specific hours were measured (Table 3). There was a significant difference in comparing the average of pain intensity between different hours of measurement in 2 groups (P < 0.001). There wasn't significant difference in comparing the average of pain intensity between two groups of Rosa damascena extract and Mefenamic acid (P = 0.22) (Figure 2). No unwanted side-effect was reported by the participants in this study.

**Table 1.** Some of the Characteristics of Participants According to Specific Groups

Groups	Rosa Damascene (N = 46)	Mefenamic Acid (N = 46)	P Value
**Age, y**	2.11 ± 22.20	2.06 ± 22.13	0.86
**BMI ^a^, kg/m ^2^**	1.99 ± 22.24	1.85 ± 21.83	0.35
**Dysmenorrhea severity (VAS ^a^) before the onset of study**	1.04 ± 6.02	1.02 ± 6.28	0.19
**Dysmenorrheal onset age, y**	1.44 ± 15.89	1.42 ±15.61	0.32
**Menarch age, y**	1.57 ± 13.26	1.42 ± 13.26	0.94

^a^ Abbreviations: BMI, Body mass index; VAS, Visual analogue scale

**Table 2.** The Average of Pain Intensity in Different Hours After the First Cycle (Before Cross-Over) in Two Groups Taking Mefenamic Acid and Rosa Damascene Capsules (N = 46)

Pain Score / Taking the Drug	Mefenamic Acid	Rosa Damascene	P value (Within Group ) ^a^	P Value (Between Group)
**Before**	6.17 ± 0.99	6.04 ± 0.98	0.25	0.35
**1 hour after**	5.37 ± 0.97	5.22 ± 0.98	0.37	-
**2 hour after**	2.91 ± 1.07	2.50 ± 0.93	0.19	-
**3 hour after**	0.98 ± 0.08	0.72 ± 0.07	0.70	-
**6 hour after**	2.13 ± 0.95	2.15 ± 0.94	0.42	-
**12 hour after**	1.48 ± 0.72	0.89 ± 0.06	0.32	-
**24 hour after**	0.85 ± 0.06	1.20 ± 0.83	0.11	-
**48 hour after**	0.30 ± 0.04	0.07 ± 0.02	0.43	-
**72 hour after**	0.00	0.00	-	-

^a^U Mann Whitney test

**Table 3.** The Average of Pain Intensity in Different Hours After the Second Cycle (After Cross-Over) in Two Groups Taking Mefenamic Acid and Rosa Damascene Capsules (N = 46)

Pain Score / Taking the Drug	Mefenamic Acid	Rosa Damascene	P-value (Within Group ) ^a^	P-value (Between Group)
**Before**	6.20 ±1.02	6.04 ± 1.01	0.45	0.22
**1 hour after**	5.43 ± 0.93	5.28 ± 0.98	0.27	-
**2 hour after**	3.09 ± 0.91	2.59 ± 0.90	0.15	-
**3 hour after**	0.98 ± 0.08	0.76 ± 0.06	0.31	-
**6 hour after**	2.22 ± 0.81	2.20 ± 0.80	0.11	-
**12 hour after**	1.91 ± 0.96	1.87 ± 0.93	0.39	-
**24 hour after**	1.15 ± 0.72	1.39 ± 0.82	0.25	-
**48 hour after**	0.37 ± 0.08	0.25 ± 0.07	0.18	-
**72 hour after**	0.00	0.00	-	-

^a^U Mann Whitney test

## 5. Discussion

This study was done in order to compare the effect of Rosa damascena extract capsule with Mefenamic acid on primary dysmenorrhea. Pain intensity in identified hours was measured in groups of Rosa damascena extract and Mefenamic acid before and after the cross-over. In both of the groups, the average of pain intensity in each measurement was significantly different in relation to before and after measurement that showed both the Rosa damascene extract and Mefenamic acid were useful in decreasing the participant's pain. In other stage, the average of pain intensity in different hours between two groups of Rosa damascene and Mefenamic acid was compared that showed no significant difference. In other words, it is shown that the effect of Rosa damascena extract and Mefenamic acid was the same in decreasing menstrual pain in participants.

In recent years, different studies have been done on aqueous alcoholic extract of various parts of Rosa damascene. In studies performed by Rakhshandeh et al. hyp-notic and analgesic effects of ethanolic extract of Rose damascene were shown in animals (10, 19, 20). Hajhashemi et al. (2010) showed the analgesic and anti-inflammatory effects of hydroalcoholic extract of Rosa damascena in mice (17). In a study, Boskabady et al. (2006) investigated the relaxation effects of Rosa damascene on pig tracheal. They concluded that ethanolic extract of Rosa damascene has the effect of relaxation on the smooth muscle of trachea of animal that is comparable to effect of Theophylline (16). Mostafa-Gharabaghi etal. in 2013 showed that the prescription of ethanolic extract of the fruit of Rosa damascene before cesarean section reduces the pain intensity of surgery and the need for analgesic drugs. Their study was a double blind placebo-controlled clinical trial, 87 patients were studied in 2 groups. Patients in Group A were given rose extracts capsule and patients in Group B were given placebo capsules. After preemptive prescription of capsule A and B (15 min before anesthesia), they evaluated the pain score with visual analog scale (VAS) in various hours after surgery (18). In present study, the ethanolic extract of Rosa damascene fruit is used too.

The effect of Rosa damascene on dysmenorrhea can be found in its compounds. The components of Rosa damascene extracts are: flavonoid (10, 23) Geraniol, eugenol, Terpene, Saponin, and etc (10). Flavonoid is the common compound of Echinophora platyloba, Saliva hydrangea and Rosa damascene. Hajhashemi et al. (2000) showed the analgesic effect of flavonoid in delaying arachidonic acid metabolism of cyclooxygenase pathway in Saliva hydrangea (24) and its analgesic effect on dysmenorrhea in Echinophora platyloba has been shown by Delaram et al. (2009) (25).

Different studies have used Rosa damascene as analgesic but most of them has been done on animals. There has been found just one study on human about its analgesic effect after cesarean (18), so the studies may not be adequate about showing the side-effects of Rosa damascene or about identifying the least effective dose on human. On the other hand, pain is an abstract and complex concept that is divided into different types such as dermal, visceral, neuralgic, acute, and chronic pain according to its source. Different factors that affect the pain can influence individual's reaction to pain (26, 27), Therefore¸ medicinal herbs used in performed reseaeches may have different effects on different types of pains. Whereas the pain effects many dimensions of human's life ¸behavior and influences general and mental health¸ physical and social functions in negative direction by lapse of time (28-30). It seems necessary to carry out more researches in the field of the ways of coping with it.

It is suggested to do more extensive studies in order to achieve the minimum effective dose of the drug. Also it can be possible to survey the effect of Rosa damascene on the other symptoms of Primary dysmenorrheal. It is necessary to do long-time researches with more samples and different doses in order to be certain and ensure.

Some of the limitations of our study were: this study was done on university students who maybe aren't the representative of all of the women and the results cannot be generalized to all the women of the child-bearing age. Also some of the factors influencing pain intensity such as culture and nutrition (2, 31-33) were uncontrollable.
